# *Monthly Prices of* Substances *Used in* Pharmacy

**Published:** 1814-06

**Authors:** 


### *Monthly Prices of* SUBSTANCES *used in* PHARMACY,

At Messrs. SELWAY and HENLEY's (Chemical and Pharmaceutical Laboratory,) 35, Upper Mary-le-bone Street, Portland Place.

| | s. | d. |
|---|---|---|
| Acetum Scillæ ........... lb. | 2 | 0 |
| Abietis Resina ............ | 2 | 3 |
| Acaciæ Gummi ............ | 2 | 3 |
| ——— elect. ...... | 3 | 3 |
| ——— Pulvis ...... | 3 | 8 |
| Acidum Aceticum .... cong. | 3 | 8 |
| ——— Benzoicum ...... lb. | 6 | 0 |
| ——— Boracicum ........ | 24 | 0 |
| ——— Oxalicum ........... | 20 | 0 |
| ——— Nitricum ........... | 4 | 6 |
| ——— Nitrosum ........... | 2 | 6 |
| ——— Muriaticum ......... | 0 | 8 |
| ——— Sulphuricum ...... | 0 | 6 |
| ——— Arom. .. | 5 | 0 |
| Alcohol sp. gr. 815 M. lb. | 5 | 6 |
| Æther sulphur. sp. gr. 789 | 10 | 0 |
| ——— rectificatus sp. gr. 744 | 12 | 0 |
| Ærugo ................ lb. | 7 | 0 |
| Aloes spicatæ extractum .... | 5 | 6 |
| ——— vulgaris extractum .... | 4 | 6 |
| ——— Barbadoes ............ | 5 | 0 |
| Alumen .................. | 0 | 4 |
| ——— exsiccatum ....... | 0 | 8 |
| Ammoniæ Murias Ang. .... | 2 | 9 |
| ——— Exot. .... | 2 | 2 |
| ——— Carbonas ....... | 3 | 6 |
| ——— Nitras ........ | 5 | 6 |
| Amygdalæ Amaræ ........ | 2 | 0 |
| ——— Dulces ......... | 5 | 8 |
| Ammoniacum (Gutt.) ...... | 6 | 6 |
| ——— (Commun.) .. | 3 | 6 |
| Anthemidis Flores (com.) .. | 1 | 9 |
| ——— (opt.) .... | 2 | 0 |
| Antimonium ............... | 3 | 0 |
| Antimonii Sulphuretum .... | 1 | 0 |
| ——— Aurum. Sulph. .. | 7 | 0 |
| ——— Oxydum ........ | | |
| ——— Murias ........ | 3 | 9 |
| Antimonium Tartarizatum .. | 5 | 0 |
| Aqua Anethi ........ cong. | 3 | 0 |
| ——— Carui ............. | 3 | 0 |
| ——— Cinnamomi vera .... | 18 | 0 |
| ——— Menthæ piperitæ .... | 2 | 6 |
| ——— pulegii .... | 2 | 6 |
| ——— Sativæ ...... | 2 | 6 |
| ——— Pimentæ ............ | 2 | 9 |
| ——— Rosæ .............. | 5 | 6 |
| ——— Cassiæ .............. | 3 | 6 |
| Arsenici Oxydum ........ lb. | 1 | 0 |
| Argenti Nitras ............ | 6 | 9 |
| Assafœtida Gummi-resina .. | 6 | 9 |
| Aurantii Cortex .......... | 2 | 6 |
| Balsamum Peruvianum ...... | 33 | 0 |
| ——— Tolutanum ...... | 15 | 0 |
| Barytæ Murias ............ | 6 | 0 |
| Benzoinum elect. .......... | 11 | 0 |
| Bismuthum ............... | 3 | 0 |
| ——— Oxydum ...... | 16 | 0 |
| ——— Nitras ...... | 16 | 0 |
| Calamina præp. ........... | 0 | 3 |
| Calumbæ Radix ........... | 2 | 6 |

| | s. | d. |
|---|---|---|
| Calx ...................... | 1 | 0 |
| Cambogia opt. ........... | 9 | 6 |
| Camphora ................. | 9 | 6 |
| Canella Cortex .......... | 3 | 4 |
| Cardamomi Semina opt. .... | 12 | 0 |
| Cascarillæ Cortex ......... | 7 | 0 |
| Castoreum ............ unc. | 4 | 4 |
| Caryophilli ............... | 11 | 0 |
| Cassia Cortex ............ | 8 | 0 |
| Catechu Extractum ........ | 2 | 3 |
| Cetaceum ................. | 3 | 0 |
| Cera flava ................ | 3 | 6 |
| ——— alba ............... | 3 | 0 |
| Ceratum Cetacei .......... | 3 | 0 |
| ——— Resinæ ........... | 2 | 8 |
| ——— Calamina ........ | 3 | 0 |
| ——— Plumbi superacet. .. | 4 | 0 |
| ——— Lyttæ .......... | 4 | 0 |
| ——— Sabinæ .......... | 2 | 8 |
| ——— Plumbi Carb. .... | 3 | 0 |
| ——— Comp. .... | 3 | 8 |
| ——— Saponis .......... | 4 | 6 |
| Cinchonæ cordifoliæ Cortex (yellow) ...... | 9 | 0 |
| ——— lancifoliæ Cortex (quilled) ...... | 13 | 0 |
| ——— oblongifoliæ Cortex (red) ........ | 16 | 0 |
| Cinnamomi Cortex ........ | 16 | 0 |
| Coccus (Coccinella) ........ | 64 | 0 |
| Colocynthidis ............ | 8 | 0 |
| Copaiba ................... | 7 | 0 |
| Colchici Radix ........... | 3 | 7 |
| Croci stigmata ............ | 64 | 0 |
| Cupri sulphas ............ | 1 | 0 |
| Cuprum ammoniatum ...... | 6 | 0 |
| Curcuma Pulvis .......... | 2 | 6 |
| Cuspariæ Cortex .......... | 4 | 0 |
| Confectio Aromatica ........ | 8 | 6 |
| ——— Aurantii ........ | 2 | 9 |
| ——— Opii ............ | 4 | 6 |
| ——— Rosæ Gallicæ .... | 1 | 10 |
| ——— Caninæ .... | 2 | 0 |
| ——— Scammoniæ ...... | 15 | 0 |
| ——— Sennæ .......... | 2 | 0 |
| ——— Amygdalæ ...... | 5 | 0 |
| Emplastrum Ammoniaci .... | 8 | 0 |
| ——— Cumini ........ | 2 | 0 |
| ——— Galbani Comp. .. | 2 | 6 |
| ——— Lyttæ ......... | 6 | 6 |
| ——— Opii ......... | 2 | 6 |
| ——— Plumbi ....... | 1 | 6 |
| ——— Resinæ ......... | 1 | 6 |
| ——— Roborans ...... | 2 | 0 |
| ——— Saponis ........ | 2 | 6 |
| ——— Hydrargyri .... | 2 | 6 |
| ——— cum Amm. | 6 | 0 |
| Extractum Anthemidis .. unc. | 1 | 0 |
| ——— Belladonnæ ...... | 1 | 0 |
| ——— Cinchonæ ...... | 3 | 0 |
| ——— resinos. | 4 | 0 |

| | s. | d. |
|---|---|---|
| Extractum Colocynthidis | 2 | 0 |
| —————— comp. | 1 | 0 |
| ——— Conii | 0 | 6 |
| ——— Gentianæ | 0 | 5 |
| ——— Glycyrrhizæ .. lb. | 3 | 0 |
| ——— Cascarillæ .... unc. | 3 | 0 |
| ——— Hæmatoxyli | 0 | 5 |
| ——— Humuli | 0 | 9 |
| ——— Hyoscyami | 1 | 0 |
| ——— Jalapæ 1*s*. 6*d*. Res. | 2 | 8 |
| ——— Opii aquos | 3 | 6 |
| ——————— resinos. | 3 | 0 |
| ——— Papaveris | 0 | 6 |
| ——— Rhæi | 2 | 4 |
| ——— Sarsaparillæ | 0 | 10 |
| ——— Taraxaci | 0 | 6 |
| Ferri carbonas ........... lb. | 0 | 9 |
| —— sulphas | 1 | 0 |
| Ferrum Ammoniatum | 3 | 6 |
| ——— Tartarizatum | 4 | 0 |
| Galbani Gummi-resinæ | 7 | 6 |
| Gentianæ Radix | 1 | 0 |
| Guaiaci Resina | 6 | 0 |
| Hydrargyrus purificatus | 5 | 6 |
| ———— cm Creta | 4 | 0 |
| Hydrargyri Oxymurias | 8 | 0 |
| ——— Submurias | 8 | 6 |
| ——————— Hydros. | 10 | 0 |
| ——— Nitrico-Oxydum .. | 8 | 6 |
| ——— Oxydum Cinereum | 16 | 0 |
| —————— Rubrum | 5 | 0 |
| ——— Sulphuretum Nig. | 3 | 6 |
| ——————— Rub. | 8 | 0 |
| ——— Præcipitatus Alb. | 8 | 0 |
| Hellebori nigri Radix | 2 | 6 |
| Ipecacuanhæ Radix | 22 | 0 |
| ———— Pulvis | 23 | 0 |
| ———— Compos. Pulv. lb. | 8 | 0 |
| Jalapæ Radix | 6 | 0 |
| ——— Pulvis | 6 | 6 |
| Kino | 12 | 0 |
| Liquor Plumbi Acetatis M. lb. | 1 | 0 |
| ——— Ammoniæ .... P.L. | 2 | 8 |
| ——— Arsenicalis | 2 | 0 |
| ——— Potassæ | 0 | 9 |
| ——— Vol. Corn. Cerv. | 1 | 3 |
| Linimentum Camphoræ comp. | 5 | 6 |
| ———— Saponis comp. .. | 5 | 6 |
| Lichen Islandicus ...... P. lb. | 1 | 9 |
| Lyttæ | 13 | 6 |
| Magnesia | 8 | 6 |
| ——— Carbonas | 3 | 0 |
| ——— Sulphas | 1 | 3 |
| Manna optima | 8 | 6 |
| ——— communis | | |
| Mel Rosæ | 2 | 6 |
| —— Despumat | 3 | 0 |
| Mices Moschatæ | 25 | 0 |
| Moschus pod, 24*s*. in gr. unc. | 40 | 0 |
| Mastiche ............... lb. | 5 | 6 |
| Myristicæ Nuclei | 25 | 0 |
| Myrrha elect. | 6 | 6 |
| Olibanum | 3 | 0 |
| Opoponax | 23 | 0 |
| Opium (Turkey) | 34 | 0 |

| | s. | d. |
|---|---|---|
| Opium (East India) ...... lb. | 32 | 0 |
| Oleum Amygdale ...... lb. | 5 | 4 |
| —— Anisi .......... unc. | 2 | 6 |
| —— Anthemidis | 5 | 0 |
| —— Cinnamomi Cassiæ | 8 | 0 |
| —— Caryophili | 5 | 0 |
| —— Cajuputi | 8 | 0 |
| —— Carui | 1 | 3 |
| —— Juniperi | 0 | 9 |
| —— Lavandulæ | 4 | 0 |
| —— Lini .......... cong. | 8 | 6 |
| —— Menthæ piperitæ unc. | 4 | 6 |
| ——————— viridis | 3 | 0 |
| —— Olivæ opt. cong. | 30 | 0 |
| ———— secundum | 18 | 0 |
| —— Origani .......... unc. | 2 | 0 |
| —— Pimentæ | 4 | 6 |
| —— Ricini optim. (per bottle) | 10 | 0 |
| ———— secund. | 7 | 0 |
| —— Rosmarini ...... unc. | 0 | 9 |
| —— Succini .. 2*s*. 4*d*. rect. | 2 | 6 |
| —— Sulphuratum ...... lb. | 1 | 6 |
| —— Terebinthinæ rectif. .. | 2 | 0 |
| Oxymel Scillæ | 3 | 0 |
| Papaveris Capsulæ (per 100) | 2 | 4 |
| Plumbi Carbonas ........ lb. | 0 | 8 |
| ——— Superacetas | 2 | 2 |
| ——— Oxydum semi-vitreum | 0 | 6 |
| Potassa Fusa .......... unc. | 0 | 6 |
| ——— cum Calce | 1 | 6 |
| Potassæ Nitras .......... lb. | 1 | 0 |
| ——— Acetas | 8 | 0 |
| ——— Subcarbonas | 1 | 2 |
| ——— Carbonas | 4 | 0 |
| ——— Sulphas | 1 | 0 |
| ——— Sulphuretum | 1 | 6 |
| ——— Supersulphas | 3 | 0 |
| ——— Tartras | 3 | 0 |
| ——— Supertartras | 2 | 0 |
| Pilulæ Hydrargyri | 6 | 0 |
| ——— Aloes comp. | 8 | 0 |
| ————— cum Colocynth. | 16 | 0 |
| ——— Saponis cum Opio .. | 18 | 6 |
| ——— Scillæ Compos. | 6 | 6 |
| ——— e Styrace | 48 | 0 |
| Piper longum | 2 | 6 |
| —— Nigrum .......... lb. | 2 | 6 |
| Pimento .............. lb. | 2 | 6 |
| Pulvis Aloes cum Canella .. | 6 | 6 |
| ———— Compositus | 10 | 0 |
| —— Antimonialis | 7 | 0 |
| —— Cinnamomi Comp .. | 16 | 0 |
| —— Cornu cervi cum Opii | 16 | 0 |
| —— Cretæ Composit. | 8 | 0 |
| ————— cum Opii | 9 | 0 |
| —— Kino Compositus | 12 | 0 |
| —— Scammoniæ Comp. unc. | 2 | 6 |
| —— Sennæ Composit. .. lb. | 8 | 0 |
| —— Tragacanthæ Comp. .. | 5 | 0 |
| Resina Flava | 0 | 5 |
| ——— Nigra | 0 | 5 |
| Rosæ Folia | 10 | 6 |
| Ras Quassiæ | 1 | 6 |
| Rhæi Radix (Russia) | 30 | 0 |
| ——— (East India) | 14 | 0 |

| | s. | d. |
|---|---|---|
| Rhæi Radix Pulvis | 15 | 0 |
| Sapo (Spanish) | 2 | 6 |
| Sarsaparilla Radix Inc. | 5 | 0 |
| Scammoniæ Gum-Resina unc. | 2 | 6 |
| Scilla recens | 1 | 3 |
| —— siccata | 5 | 0 |
| ———— pulvis | 6 | 0 |
| Senegæ Radix | | |
| Sennæ Folia Opt. Alexand. | 6 | 6 |
| Serpentariæ Radix | 30 | 0 |
| Simaroubæ Cortex | 4 | 0 |
| Sodæ Boras | 4 | 6 |
| —— Carbonas | 7 | 0 |
| —— Subcarbonas | 1 | 4 |
| ———— exsiccata | 2 | 8 |
| Sinapis super. | 2 | 6 |
| —— secund. | 2 | 0 |
| Sodæ Sulphas | 0 | 8 |
| —— Phosphas | 2 | 8 |
| —— Acetas unc. | 0 | 10 |
| Soda tartarizata lb. | 2 | 4 |
| Spongia ustæ unc. | 1 | 3 |
| Spiritus Ammoniæ M. lb. | 4 | 0 |
| ———— aromaticus | 4 | 6 |
| ———— fœtidus | 3 | 6 |
| ———— succinatus | 4 | 6 |
| —— Cinnamomi ver. | 3 | 6 |
| —— Juniperi Compos. | 3 | 0 |
| —— Lavandulæ | 4 | 6 |
| ———— Compos. | 5 | 0 |
| —— Myristicæ | 3 | 0 |
| —— Pimentæ | 3 | 0 |
| —— Rosmarini | 3 | 6 |
| —— Ætheris Aromaticus | 6 | 0 |
| ———— Compositus | 6 | 0 |
| ———— Nitrici sp. gr. 860 | 5 | 6 |
| ———— Sulphurici | 6 | 0 |
| —— Vini rectificatus | 28 | 0 |
| Syrupus Aurantii | 1 | 8 |
| —— Rutæ | 1 | 8 |
| —— Papaveris | 2 | 0 |
| —— Violæ | 2 | 10 |
| —— Rhei | 2 | 10 |
| —— Rhamni | 2 | 0 |
| —— Croci | 3 | 0 |
| —— Tolutanea | 2 | 0 |
| —— Rheados | 2 | 0 |
| Sulphur | 0 | 6 |
| —— Lotum | 1 | 0 |
| —— Præcipitatum | 1 | 0 |
| Tamarindus opt. | 2 | 6 |
| Terebinthina Vulgaris | 0 | 9 |
| ———— Canadensis | 8 | 0 |
| ———— Chia | 6 | 0 |
| Tragacantha Gummi | 10 | 0 |
| ———— Pulvis | 10 | 6 |
| Tinctura Aurantii M. lb. | 3 | 9 |
| —— Aloes | 4 | 0 |
| ———— Composita | 9 | 6 |
| —— Assafœtidæ | 5 | 6 |
| —— Balsa. Tolut. | 5 | 6 |
| —— Benzoini Comp. | 6 | 9 |
| —— Camphoræ Comp. | 4 | 0 |

| | s. | d. |
|---|---|---|
| Tinctura Cardamomi | 4 | 6 |
| ———— Comp. | 4 | 0 |
| —— Calumbæ | 3 | 9 |
| —— Capsici | 3 | 9 |
| —— Cascarillæ | 4 | 6 |
| —— Castorei | 7 | 0 |
| —— Catechu | 4 | 0 |
| —— Cinchonæ | 6 | 0 |
| ———— Compos. | 6 | 0 |
| —— Cinchonæ Ammon. | 7 | 0 |
| —— Croci | 6 | 6 |
| —— Digitalis | 4 | 6 |
| —— Ferri Ammoniati | 4 | 0 |
| —— Cinnamomi | 4 | 6 |
| ———— Comp. | 4 | 6 |
| —— Gentianæ Comp. | 4 | 0 |
| —— Guaiaci | 6 | 0 |
| ———— Ammon. | 6 | 0 |
| —— Hellebori Nigri | 4 | 6 |
| —— Humuli | 5 | 0 |
| —— Hyoscyami Nig. | 4 | 6 |
| —— Jalapæ | 4 | 6 |
| —— Japonica | 4 | 6 |
| —— Ferri Muriatis | 5 | 0 |
| —— Kino | 5 | 0 |
| —— Lyttæ | 3 | 9 |
| —— Myrrhæ | 4 | 6 |
| —— Opii | 7 | 0 |
| ———— Camphorat. | 4 | 6 |
| —— Quassiæ | 3 | 9 |
| —— Rhei | 4 | 6 |
| ———— Comp. | 4 | 0 |
| —— Scillæ | 4 | 6 |
| —— Sennæ | 4 | 9 |
| —— Serpentariæ | 6 | 0 |
| —— Valerianæ | 4 | 6 |
| ———— Amm. | 5 | 6 |
| —— Zingiberis | 4 | 6 |
| Valerianæ Radix | 1 | 8 |
| Veratri Radix | 1 | 6 |
| Unguentum Hydrarg. Mit. | 3 | 0 |
| ———— Nitratis | 3 | 3 |
| ———— Nitrico-oxy. | 2 | 9 |
| ———— Sulphuris Comp. | 2 | 0 |
| ———— Sambuci | 2 | 0 |
| ———— Virid | 1 | 8 |
| ———— Altheæ | 2 | 0 |
| ———— Cetacei | 3 | 6 |
| ———— Veratri | 1 | 8 |
| ———— Zinci | 2 | 0 |
| ———— Hyd. præcip. Alb. | 3 | 0 |
| ———— Picis | 1 | 6 |
| Uvæ Ursi Folia | 2 | 4 |
| Vinum Aloes | 3 | 6 |
| —— Antimoniale | 3 | 0 |
| —— Ferri | 3 | 6 |
| —— Ipecacuanhæ | 5 | 6 |
| —— Opii | 8 | 0 |
| Zinci Oxydum | 4 | 6 |
| —— Sulphas purif. | 2 | 0 |
| —— Acetas unc. | 1 | 6 |
| Zincum lb. | 1 | 6 |
| Zingiberis Radix opt. | 2 | 4 |

Lisbon Leeches, per dozen, 3s. 6d.—Bourdeaux Leeches, 4s.

French ditto, true, 7s.—English ditto, true, 12s.

